# Biological Investigation of 2-Thioxo-benzo[g]quinazolines against Adenovirus Type 7 and Bacteriophage Phi X174: An In Vitro Study

**DOI:** 10.3390/cimb45050244

**Published:** 2023-04-28

**Authors:** Hatem A. Abuelizz, Ahmed H. Bakheit, Mohamed Marzouk, Waled M. El-Senousy, Mohamed M. Abdellatif, Essam E. Ali, Gamal A. E. Mostafa, Rashad Al-Salahi

**Affiliations:** 1 Department of Pharmaceutical Chemistry, College of Pharmacy, King Saud University, Riyadh 11451, Saudi Arabia; 2 Chemistry of Tanning Materials and Leather Technology Department, Organic Chemicals Industries Division, National Research Centre, Dokki, Cairo 12622, Egypt; 3 Environmental Virology Laboratory, Water Pollution Research Department, Environment and Climate Change Research Institute and Food-Borne Viruses Group, Centre of Excellence for Advanced Sciences, National Research Centre (NRC), 33 El-Buhouth Street, Dokki, Giza 12622, Egypt; 4 Department of Chemistry, Graduate School of Science, Tokyo Metropolitan University, 1-1 Minami Osawa, Tokyo 192-0397, Japan

**Keywords:** benzo[g]quinazolines, bacteriophage phiX174, adenovirus type 7, docking study, cytotoxicity

## Abstract

Mortality and morbidity caused by viruses are a global health problems. Therefore, there is always a need to create novel therapeutic agents and refine existing ones to maximize their efficacy. Our lab has produced benzoquinazolines derivatives that have proven effective activity as antiviral compounds against herpes simplex (HSV 1 and 2), coxsackievirus B4 (CVB4), and hepatitis viruses (HAV and HCV). This in vitro study was aimed at investigating the effectiveness of benzoquinazoline derivatives **1**–**16** against adenovirus type 7 and bacteriophage phiX174 using a plaque assay. The cytotoxicity against adenovirus type 7 was also performed in vitro, using a MTT assay. Most of the compounds exhibited antiviral activity against bacteriophage phiX174. However, compounds **1**, **3**, **9**, and **11** showed statistically significant reductions of 60–70% against bacteriophage phiX174. By contrast, compounds **3**, **5**, **7**, **12**, **13**, and **15** were ineffective against adenovirus type 7, and compounds **6** and **16** had remarkable efficacy (50%). Using the MOE-Site Finder Module, a docking study was carried out in order to create a prediction regarding the orientation of the lead compounds (**1**, **9**, and **11**). This was performed in order to investigate the activity of the lead compounds **1**, **9**, and **11** against the bacteriophage phiX174 by locating the ligand–target protein binding interaction active sites.

## 1. Introduction

Benzoquinazolines, fundamental components of numerous well known heterocyclic systems, have emerged as a vital building block in the synthesis of numerous therapeutically relevant compounds [1]. Studies on the antiviral activity of benzoquinazolines have led to a plethora of studies on benzoquinazolines and their myriad pharmacological effects [1]. In vitro screening of 3-benzyl (phenethyl)benzo[g]quinazoline derivatives for anti-hepatitis A virus (HAV) activity, utilizing the cytopathic effect inhibition assay, revealed significant anti-HAV activity, especially when compared to the standard treatment drug amantadine [2]. Furthermore, benzo[g]quinazoline’s ability to inhibit picornaviral 3C protease (human rhinovirus 3Cpro) has been investigated. Other than having significant inhibitory effects, it may also provide insight into its powerful antiviral efficacy. In addition, benzyl and methyl compounds of benzoquinazolines have been proven to be more efficient inhibitors of HAV-3C protease than telaprevir. The reemergence of drug-resistant virus strains is what initially inspired the development of antiviral drugs [3]. Herpes simplex viruses (HSV-1 and 2) and coxsackievirus 4 (CVB4) are vulnerable to a number of benzo[g]quinazoline derivatives, some of which have demonstrated potent antiviral activity [4]. It has also been found that certain benzoquinazolines significantly impede the development of the influenza H1N1 and H5N1 virus strains that were investigated [5]. With the ongoing need to find new antivirals, we chose to investigate benzoquinazoline’s efficacy against adenovirus type 7 and bacteriophage phiX17.

PhiX174 belongs to the *Microviridae* bacteriophage family. It has a tiny, icosahedral, single-stranded DNA, and it is non-tailed [6,7,8,9]. Several model systems have been used, among them *Microviridae* for genome engineering, icosahedral virus packaging, and virus infection mechanisms [8,10,11]. Bacteriophage PhiX174 has been employed in a significant number of experiments and can be used as a marker in aquatic settings to signify viral or fecal contamination because it is a coliphage (International Organization for Standardization, ISO 10705-2), and its 5386-nucleotide genome and nonpathogenic status explained that [7,12,13]. Suzuki et al. reported, in 1974, that, unlike phage adsorption, bacteriophage PhiX174 DNA replication can be sustained by a variety of *Escherichia coli* (*E. coli*) strains and distantly related bacteria, such as *Pseudomonas aeruginosa* [7,14]. This was despite the fact that phage adsorption is only possible in bacteria that have a specific receptor. *The Microviridae* bacteriophage, illustrated by the model phiX174 phage, and its *E. coli* hosts, are important components of a healthy human gut microbiota. Although much has been learned from studying bacteriophage phiX174 over the past half-century, the *E. coli* host response to infection has never been thoroughly examined until now. Rapid adaptability to novel hosts is crucial for the propagation of emerging viral infections [15].

**Figure 1:**
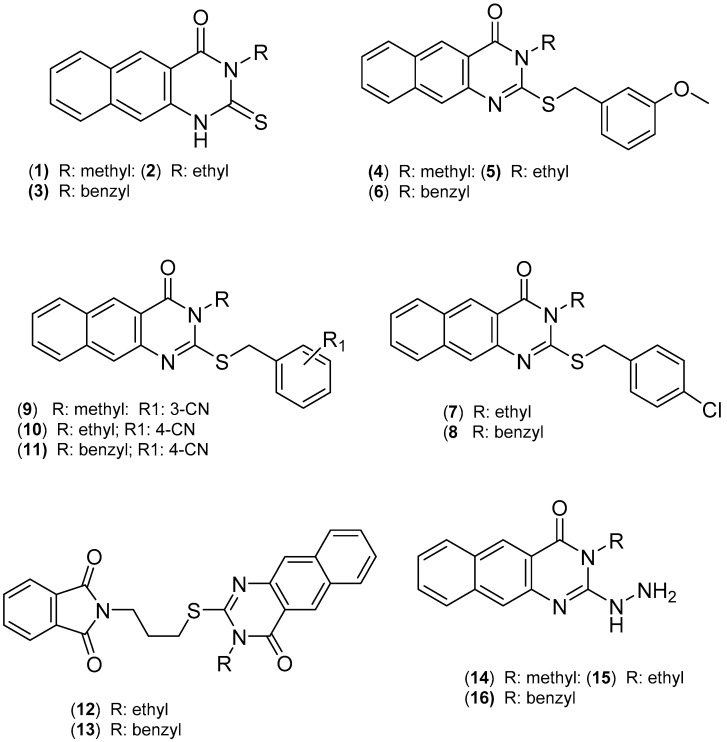
The synthesized benzo[g]quinazolines derivatives (**1**–**16**).

Adenovirus type 7 is an icosahedral *Adenoviridae* virus with 30,000–42,000 nucleotides of straight, double-stranded DNA and no envelope [16,17]. In immunocompromised and nonimmune impaired hosts, it causes pneumonia and disseminated infection, respectively. Human adenoviridae are classified into six serotypes based on shared hemagglutination and DNA sequences (A–F) [18]. Adenovirus type 7 can be recognized in Subgroup B. Subgroup B Ad, particularly species B 1 with Adenovirus type 7, causes severe lower respiratory tract infections in humans [18]. Acute Adenovirus Type 7 pneumonia killed 22–39% of young children in developing countries, where the death risk is higher. As a result, Adenovirus type 7 is a deadly illness, especially for newborns and adults in crowded environments [18]. Virus infections are a significant problem for contemporary remediation. This is especially true for illnesses brought on by rare but potentially severe viruses. Due to their high rates of asymmetric mutation, these viruses bypass the immune system and are resistant to conventional antiviral drugs. Commercial drugs that are currently available only target the symptoms, not the underlying infection, and it is common knowledge that there is no therapy or cure for the viruses. Our extensive laboratory studies of benzoquinazolines have shown that these compounds have potent pharmacological effects in a range of contexts [1,2,3,4,19,20,21,22,23,24,25,26,27], including against a variety of viral infections. As stated above, our results guide the planning of future investigations and pave the way for the discovery and development of benzo[g]quinazolines as antiviral agents against adenovirus type 7 and bacteriophage phiX174. Thus, we performed this work to determine the effectiveness of a new 2-thioxo-benzoquinazolines (Figure 1) against adenovirus type 7 and bacteriophage phi X174. The MOE-Site Finder Module was used to investigate the active sites of the DNA bacteriophage X174 protein, and molecular docking was used to model the interaction between a small molecule and a protein at the atomic level, allowing us to describe the behavior of small molecules in the binding sites of target proteins and comprehend fundamental biochemical processes.

## 2. Materials and Methods

### 2.1. The Target 2-Thioxo-benzo[g]quinazoline (***1***–***16***)

Compounds **1**–**16** were previously synthesized and properly documented [2,3,20,23,26]. Analytical tools, such as NMR, IR, and MS spectra, were used to analyze and confirm all **1**–**16** structures in great detail. The benzoquinazolines **1**–**16** were obtained as colored solid compounds. Specifically, under a refluxing environment in dimethyl formamide (DMF) for 3–5 h, either ethyl(methyl) isothiocyanate or (benzyl)isothiocyanate can react with 3-amino-2-naphthaoic acid to produce the parent intermediates **1**, **2**, and **3** in good yields (81–86%). The thioalkylated benzo[g]quinazolines **4**–**13** were produced by reacting compounds **1**–**3** with the appropriate aralkyl halide (benzyl substituted halides) at 80 °C for 20 h in the presence of a base, such as potassium carbonate (K_2_CO_3_). The hydrazine derivatives **14**–**16** were obtained by reacting **1**–**3** with hydrazine hydrate in boiling DMF for 15–18 h. Detailed accounts of these have been written up and published [2,3,20,23,26].

### 2.2. Cytotoxicity Test

The cytotoxicity experiment was carried out in accordance with the methods described by Simoes and Walum et al. [28,29]. An amount of 100 mg were dissolved in 1 mL of EtOH. Then, 1 mL of benzoquinazoline was treated with 24 μL of a 100× antibiotic-antimycotic mixture [(Penicillin-Streptomycin (antibacterial)-Amphotericin B (antifungal)]. Next, the non-toxic dose was estimated by culturing 96-well plates (Greiner-Bio one, Germany) with Hep 2cell line (obtained from the Holding Corporation for Biological Products & Vaccines, VACSERA, Egypt) and inoculating 100 μL of each dilution for 24 h. For the cytotoxicity assay, we employed an inverted light microscope to observe cell morphology and a trypan blue dye exclusion technique to measure cell viability.

### 2.3. Cell Morphology Evaluation by Inverted Light Microscopy

In 96-well tissue culture plates, Hep 2 cell line cultures (2 × 10^5^ cells/mL) were prepared independently (Greiner-Bio one, Frickenhausen, Germany). After 24 h of incubation at 37 °C in a humidified 5% (*v*/*v*) CO_2_ environment, the cell monolayers were cultured, and the medium was removed from each well and refilled with 100 μL of twofold dilutions of the tested sample, made in DMEM (GIBCO BRL). An amount of 100 μL of DMEM, without samples, was added for cell controls. In a humidified 5% (*v*/*v*) CO_2_ environment, all cultures were incubated at 37 °C for 72 h. Loss of confluence, cell rounding and shrinkage, and cytoplasmic granulation and vacuolization were detected daily. Morphological alterations were scored [29].

### 2.4. Cell Viability Assay

The trypan blue dye exclusion method was used following the documented procedure [30]. In 12-well tissue culture plates, Hep 2 cell line cultures (2 × 10^5^ cells/mL) were developed (Greiner-Bio one, Germany). Following the 24 h incubation period, 100 µL of tested sample dilutions (two-fold dilutions) were applied to each well before performing the same analysis for the tested sample as indicated above in the cell morphology evaluation. After 72 h, the medium was withdrawn, the cells were trypsinized, and an equal amount of the aqueous solution, containing 0.4% (*w*/*v*) trypan blue dye, was added to the suspension of the cells. The number of viable cells was measured using a phase contrast microscope.

### 2.5. Determination of Adenovirus Type 7 Titers (Number of Infectious Viral Particles) Using Plaque Assay

Adenovirus type 7 (1 × 10^6^ PFU/mL) was mixed with 100 μL of non-toxic dilutions. In 12 multi well plates, Hep 2 cells were inoculated with 100 μL of 10 fold dilutions of treated and untreated adenovirus type 7. Then, 1 h of adsorption at 37 °C in a 5% CO_2_ water vapor environment without rocking was performed. To prevent cell drying, plates were bounced periodically. After adsorption, 1 mL of 2× media (Dulbecco’s Modified Eagle Medium, Gibco-BRL (DMEM)) and 1% agarose were added to each well. The plates were incubated at 37 °C in a 5% CO_2_ water vapor atmosphere. The plaques were counted, following formalin fixation and 0.4% crystal violet staining. The PFU/mL virus titers were calculated [30].

### 2.6. Bacteriophage Phi X174 Quantification

Infectious bacteriophage phiX174 virus (1 × 10^5^, 1 × 10^6^, 1 × 10^7^) PFU/mL was utilized at the lowest non-toxic doses on Hep-2 cell line. The 23rd version of standard techniques for water and wastewater assessment quantified the infectious bacteriophage phiX174 virus (APHA, 2017). Bacteriophage phiX174 (ATCC 13706B1), and *Escherichia coli* C (ATCC 13,706).

### 2.7. MTT Assay

Antiviral colorimetric assay: a monolayer of Hep-2 cells was cultured in 96-well microtiter plates. Before viral infection, dilutions of the extracts, generated as described above, were applied. Each well received ten infectious doses of virus and was incubated for 48 h at 37 °C in a humidified 5% CO_2_ environment. Untreated infected cells, treated uninfected cells, and untreated uninfected cells comprised the controls. The vitality of cells was determined using the MTT colorimetric method in accordance with the Mosmann method [31]. Briefly, the supernatants were removed from the wells, and 28 µL of a 2 mg/mL MTT (Sigma, St. Louis, MO, USA) solution in PBS were added to each well. After incubating the plates for 1.5 h at 37 °C, 130 µL of DMSO-d_6_ was added to dissolve the MTT crystals. The plates were shaken for 15 min, and the optical density at 492 nm (OD492) was measured using a multiwall spectrophotometer. The 50% cytotoxic concentration (CC_50_) of the test extract was defined as the concentration that reduced the OD492 of uninfected cells treated with the extract to 50% of that of untreated uninfected cells. The protection percentage was computed as [(A − B)/(C − B)] 100, where A, B, and C were the OD492 of treated infected cells, untreated infected cells, and untreated uninfected cells, respectively. The CC_50_ values for each substance were derived from dose–response curves. The CC_50_ is the averages of four experiments with five concentrations within the drug’s inhibitory range.

### 2.8. Molecular Docking

Three-dimensional structure and molecular docking investigation were both created in the Molecular Operating Environment (MOE) programme [3]. The MOE-Dock docking software was used, and the MOE-implemented ligplot was utilized to show the protein–ligand interaction. The active site of the bacteriophage phix174 protein, which serves as the target, was specified using a co-crystallized ligand atom.

### 2.9. Statistics

On the Hep-2 cell line, the results of cytotoxicity experiments on the examined materials were confirmed by five repetitions. This number of repetitions was conducted for cytotoxicity testing by cell morphology analysis or cell viability assay. When comparing the antiviral activity of various materials against adenovirus type 7 or bacteriophage phi X174, the same number of repetitions was used. This number of repetitions was used for adenovirus type 7 or bacteriophage phi X174 virus plaque assays. Mean and standard deviation were calculated for non-toxic concentrations, adenovirus type 7 or bacteriophage phi X174 virus reductions. A data set’s mean $\bar{x}$ is the sum of all the data divided by the number n.

(1) $$s=\sqrt{\frac{1}{N-1}\sum_{i=1}^{N}{\left(x_{i}-\bar{x}\right)}^{2}},$$

where, *x_i_* is one sample value, $\bar{x}$ is the sample mean, and *N* is the sample size.

This is easily calculated by a mean and standard deviation calculator (https//www.calculator.net/standard-deviation-calculator.html, accessed on 23 April 2023). Using linear regression analysis, the CC50 for adenovirus type 7 was estimated. The data were analysed using Graph Pad Prism version 6.0 software.

## 3. Results and Discussion

### 3.1. Biological Evaluation

The antiviral capabilities of the synthesized benzoquinazolines derivatives were developed [2,3,4]. As a result of this study, the antiviral activity of (**1**–**16**) was assessed in vitro against adenovirus type 7 and bacteriophage phiX174. The Hep-2 cell line exhibited some resistance to the toxicity of the investigated **1**–**16**, as shown by cytotoxicity testing. With a higher concentration of tested chemicals on the Hep-2 cell line, the absence of in vitro toxicity symptoms, such as cell death, rounding, shrinkage, etc., was evident. This could relate to the nature, origin, structure, and form of the cell line. The fact that the Hep-2 cell line is derived from human epidermoid larynx carcinoma may explain why it possesses distinct capabilities against hazardous substances [32]. This was validated by microscopy, trypan blue, and the MTT assay, and the CC_50_ values of suggested that the Hep-2 cell line was resistant to the toxicity of the investigated benzoquinazolines. Some of the non-toxic doses (Table 1) of the examined materials showed antiviral activity against human adenovirus type 7 with variable efficiencies. Most benzoquinazolines derivatives **1**–**16** have proven antiviral activity, but their efficacy is varied. Due to the unique virus structure, size, and genetics, this observation was expected. Despite the DNA genomic structure of adenovirus type 7 and bacteriophage phiX174, benzoquinazolines derivatives have produced varied results. It is conceivable that only the compounds’ direct action on the virus, not their ability to prevent viral adsorption or disrupt the viral reproduction cycle, was employed to assess viral infectivity. This may be because the investigations only target the virus with non-toxic amounts of the examined compounds. This study’s results may have originated from any of the pathways above or from the compounds studied at non-toxic levels that impacted the genome. Varying cell lines revealed no toxicity at different doses. Due to the fact that not all of the tested materials exhibited the same antiviral potency against bacteriophage phiX174 in relation to adenovirus type 7, it is difficult to develop a safe, easy-to-obtain, and propagating viral model, as a bacteriophage expresses the antiviral effect of certain materials against human enteric and respiratory viruses. This may be related to differences in host nature, structure, and type. All of the studied benzoquinazolines derivatives, **1**–**16**, exhibited varied levels of activity against bacteriophage phiX174. It was observed that the reduction activities ranged from low to high (Table 2). Compound **7** appeared to be inactive, and compounds **4** and **5** have shown the lowest reduction activity of 10%. However, compounds **1**, **2**, **3**, **9**, and **11** have demonstrated the highest antiviral efficacy, with reduction percentages ranging from 50% to 70%. However, the benzoquinazolines derivatives **8**, **10** and **12**–**16** have shown moderate effects, ranging between 20% to 40%. On the other hand, benzoquinazolines derivatives **6** and **16** have demonstrated good efficacy, with a 50% reduction against adenovirus type 7. Furthermore, compounds **1** and **8** have shown moderate reduction effects of 33% and 40%, respectively. However, benzoquinazolines derivatives **3**, **5**, **7**, **12**, **13**, and **15** were inactive. In addition, Table 3 summarizes the CC_50_ values obtained by assessing the benzoquinazolines **1**–**16** in vitro for their cytotoxicity against Hep 2 using MTT tests. Various hypotheses, regarding the mechanisms of the targets’ actions, have been proposed. Actually, among the investigated active compounds, perhaps one displays desirable binding capabilities with the DNA virus genome. It is necessary to conduct additional research in order to determine which particular fraction of the promising compounds is responsible for the antiviral potency of those compounds or can be utilized to illustrate the synergism of the constituents that relate to the antiviral potency of the materials that have been studied.

Using the aforementioned findings, the basic structural activity relationship (SAR) has been established. The parent compound **1**, which has a methyl substitution at position 3 in the benzo[g]quinazoline platform, has the highest reduction percentage of 70%, followed by compounds **9** and **11**, with reduction percentages of 66.7% and 56.7%, respectively. The activity profile was not improved by converting the thioxo group in compound **1** to the thioether group in compound **4** or the hydrazine derivative in compound **14**. However, the activity was positively enhanced by compound **9**. The chemical transformation of the reduction-active parent **3** into compound **11** resulted in a 56.7% increase in activity against bacteriophage phix174. This was due to the substitution of benzyl at position 3 in the benzoquinazoline platform. Moreover, the antiviral activity was found to differ according to the type of substitution. For instance, compound **9**, which contains a cyano group in the benzyl molecule’s meta position, is more effective against bacteriophage phix174 than compounds **10** and **11**, which include the same substituent in the para position. The biological effects of substituted methoxy- benzoquinazoline derivatives **4**, **5**, and **6** are different; compound **6** with N-benzyl substitution was more active than compounds **4** and **5** with N-ethyl and methyl substitution, providing additional support for the reported fact about the lipophilicity feature of the benzyl group [1]. Similarly, compound **8**, with a chloro substitution at the benzyl group, was more active than compound **7** due to an N-benzyl substitution. Additional chemical changes on the thioxo group yielded derivatives **14**, **15**, and **16**, although only derivative **16** showed a higher reduction activity of 50% against adenovirus type 7, whereas against bacteriophage phiX174, it has shown no improvement in the activity (20%). Thus, adding an electron-donating or electron-withdrawing group to the benzyl group or inserting a hydrogen-rich group resulted in a wide range of different activity profiles.

### 3.2. Docking Study

The “MOE-Site Finder” Module, which determines putative recognition sites from the protein’s three-dimensional atomic coordinates, was used to investigate the active sites of the DNA bacteriophage PhiX174 protein. Given that no energy models were used in its development, the Site Finder module is classified as a geometric approach. Instead, a general categorization of chemical classes was attempted, with an emphasis on the locations and accessibility of atoms within proteins. After determining these areas, in Figure 1, phantom atoms were placed there to facilitate the molecule docking computation at the desired locations. Benzoquinazolines derivatives **1**, **9**, and **11** were included in the investigation. All these compounds were designed in Marvin Sketch (Marvin was used for drawing, displaying, and characterizing chemical structures, substructures, and reactions, Marvin version 21.19.0 (Internal build ID: 21.19.0-13698), hem Axon (https://www.chemaxon.com, accessed on 23 April 2023)), exported to mol files, and then the energy was minimized in MOE with the default settings.

**Figure 2:**
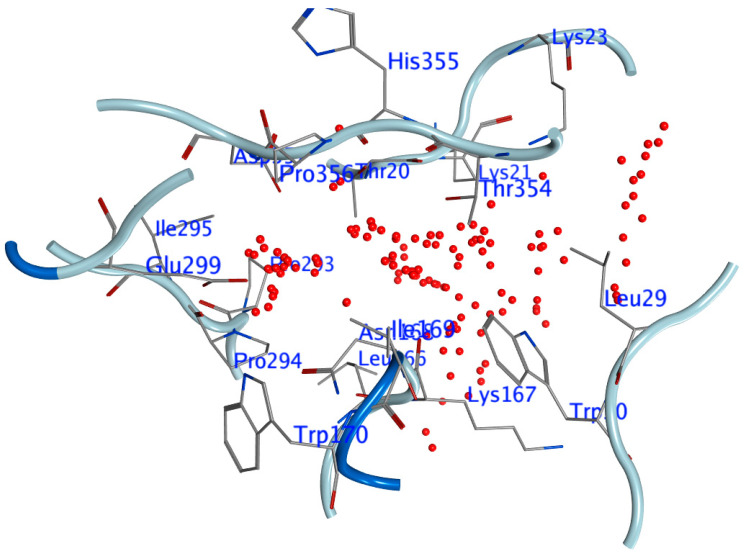
Predicted binding pocket of the bacteriophage phiX174 by means of molecular operating environment (MOE) site finder.

Due to the lack of studies on this topic, this research sought to investigate the ligand–target protein binding interaction in bacteriophage phiX174. In order to identify the binding interactions, molecular docking was carried out using MOE-dock with the majority of the default tools. The crystal structure of bacteriophage phiX174 revealed a ligand bound into the target site. As illustrated in Figure 2, the crystal structure of the bacteriophage phiX174, which was used in conjunction with the Site-Finder Module to predict the ligand-binding site, revealed that F: (LYS64 CYS164 HIS165 LEU166 LYS167 ASN168 ILE169 TRP170 TRP244 ARG291 PRO293 PRO294 ILE295 GLU299 ARG353 THR354 HIS355 PRO356 ASP357) and J: (GLY19 THR20 LYS21 LYS23 LYS25 GLY26 ALA27 ARG28 LEU29 TRP30 GLN36 PHE37).

Docking scores and binding interactions of the three most active compounds (**1**, **9**, and **11**), with the target protein, are summarized in Table 4. It is possible that these findings could be useful in the development of new and more effective drugs to combat bacteriophage phix174.

**Figure 3:**
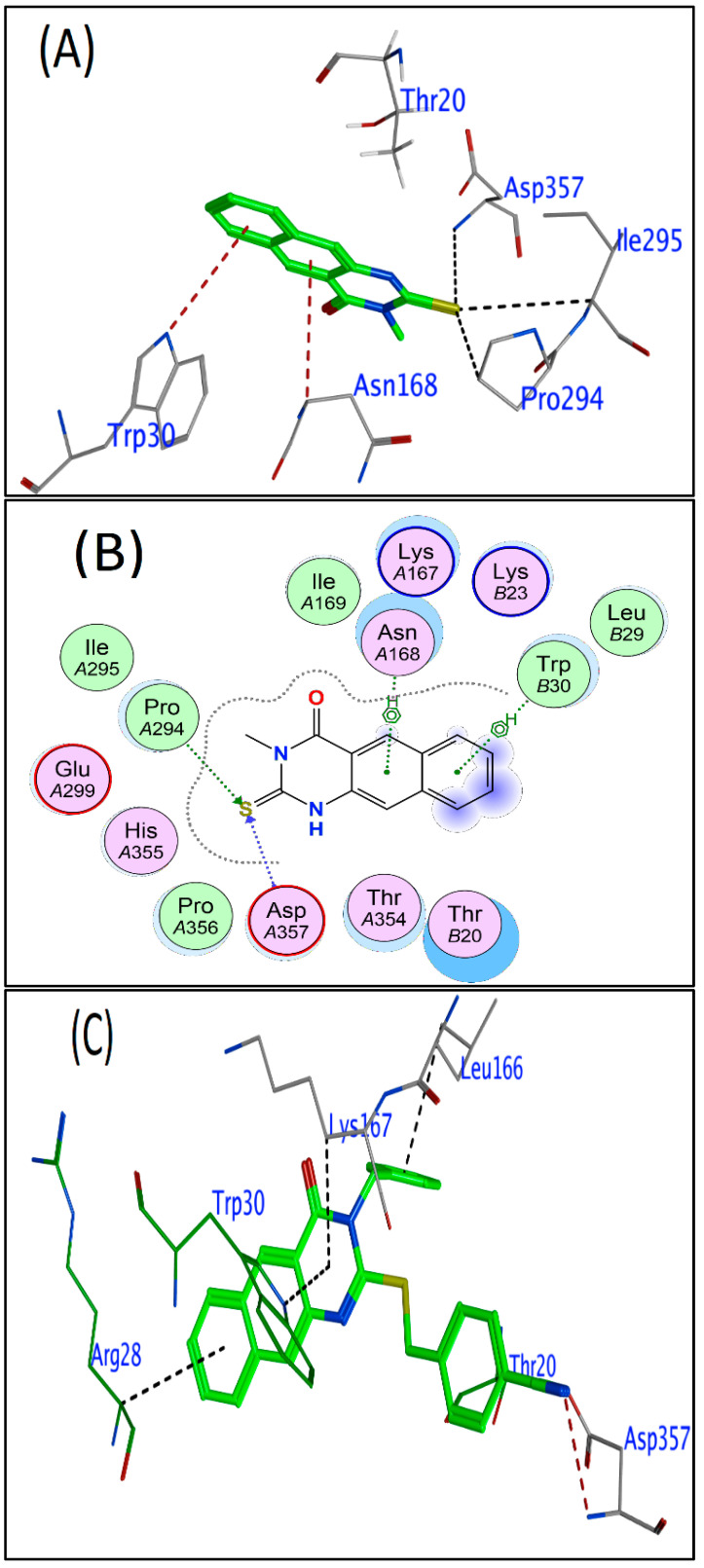

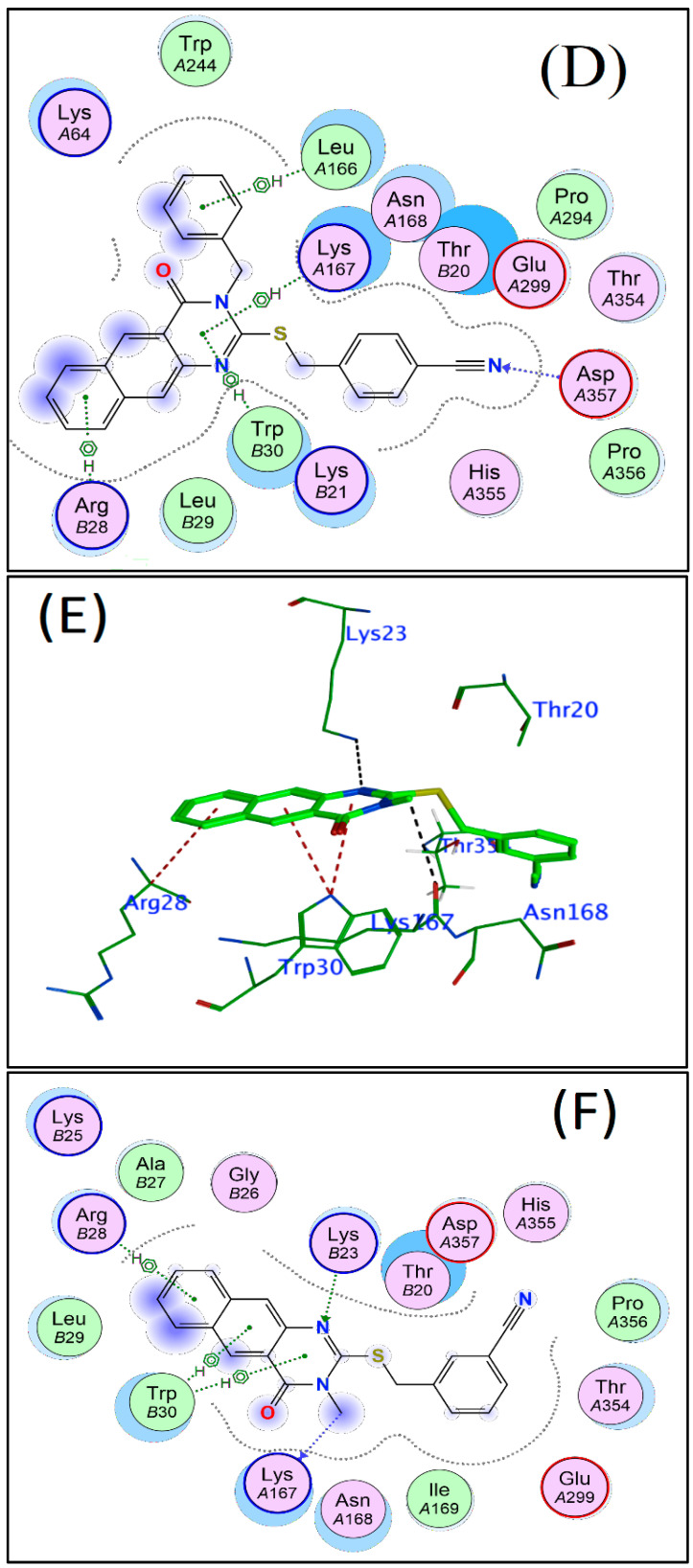
The phiX174 Ligand Interaction Diagram (2BPA). Polar residues are pink, hydrophobic residues are green, acidic residues are red, and basic residues are blue. Green and blue arrows indicate hydrogen bonding to sidechain and backbone atoms, respectively. A naphthyl icon represents a π–π stacking interaction, while a benzene with a + represents a cation–π interaction. Blue “clouds” on ligand atoms indicate the solvent-exposed surface area of ligand atoms (darker and larger clouds mean more solvent exposure). Light-blue “halos” around residues indicate the degree of interaction with ligand atoms (larger, darker halos mean more interactions). The dotted contour reflects steric room for methyl substitution. The contour line is broken if it is closest to an atom which is fully exposed. Two-dimensional and three-dimensional ligand interactions diagram for the bacteriophage phiX174 with benzoquinazoline **1** (**A**,**B**), benzoquinazoline **11** (**C**,**D**), and with benzoquinazoline **9** (**E**,**F**), respectively.

Benzoquinazolines derivatives **1**, **9**, and **11** have demonstrated the highest reduction activity against bacteriophage phiX174 (Table 2), and hence these compounds also exhibited favorable docking scores. The docking scores for compounds **1** and **9** were −5.38 and −7.27, respectively, while the value for compound **11** was −6.65. The docking score results indicate good interaction with the target protein. A total of four different amino acid residues, including PRO294 (F), ASP357 (F), ASN168 (F), and TRP30 (J), have established two hydrogen bonds and two pi-H bonds with benzoquinazoline derivative **1**, as shown in Figure 3A,B. Figure 3 depicts the ligand–receptor interaction in three-dimensional and two-dimensional space. The reducing activity against bacteriophage phiX174 in vitro corroborated these docking findings. The ligand interaction diagram in Figure 3C,D showed important interactions between the compound **9** and the ligand of the bacteriophage phix174. The thiobenzonitrile moiety (hydrophobic pocket) forms backbone hydrogen bond interactions with ASP357, and it is attracted to LYS167, and it also possesses cation–π interactions with LEU166 (F), ARG28 (J), and TRP30 (J). Compound **11** was the second most active ligand, forming two hydrogen bonds with the ligand and three pi-H bonds with LYS167 (F), LYS23 (J), ARG28 (J), and TRP30 (J), as indicated in Figure 3E,F.

## 4. Conclusions

Benzo[g]quinazolines derivatives (**1**–**16**) were evaluated for their antiviral activity against bacteriophage phiX174 and adenovirus type 7. The cytotoxicity against adenovirus type 7 was assessed using a MTT assay. Against bacteriophage phiX174, benzoquinazoline derivatives **1**, **2**, **3**, **9**, and **11** have shown the highest reduction activity, while benzoquinazolines **6** and **16** have shown a remarkable effect against adenovirus type 7. The docking findings of the most active compounds agree with the findings of the experimental in vitro data. It was discovered that a total of four different amino acid residues, including PRO294 (F), ASP357 (F), ASN168 (F), and TRP30 (J), have established two hydrogen bonds and two pi-H bonds with benzoquinazoline derivative **1**. In addition, the thiobenzonitrile moiety (hydrophobic pocket) in compound **9** forms backbone hydrogen bond interactions with ASP357, which are attracted by LYS167, as well as cation–π interactions with LEU166 (F), ARG28 (J), and TRP30 (J). Chemically transforming **3** with 50% reduced activity into **11** increased activity against bacteriophage phiX174 by 56.7%. Furthermore, compound **6**, with N-benzyl substitution, was more active than compounds **4** and **5** with N-methyl and ethyl substitution, lending confirmation to the previously stated fact concerning the benzyl group’s lipophilicity. However, further laboratory study is required to fully understand the mechanism of action of benzoquinazolines.

## Figures and Tables

**Table 1:** Non-toxic doses of tested benzoquinazolines derivatives (**1**–**16**) on Hep-2 cell line.

Comp.	Non-Toxic Doses (µg/mL)	Comp.	Non-Toxic Doses (µg/mL)
**1**	65 ± 1.8	**9**	70 ± 2.4
**2**	60 ± 1.1	**10**	50 ± 2.4
**3**	70 ± 2	**11**	65 ± 1.6
**4**	55 ± 1.6	**12**	30 ± 1.8
**5**	60 ± 2.1	**13**	45 ± 1.9
**6**	65 ± 3	**14**	60 ± 2
**7**	60 ± 2.2	**15**	55 ± 1
**8**	50 ± 2.2	**16**	65 ± 3.4

**Table 2:** Percentages of reduction performed by the non-toxic doses of compounds **1**–**16** against adenovirus type 7 and bacteriophage phiX174. Results are mean ± standard deviation of three results against three doses of virus (1 × 10^5^–1 × 10^6^) PFU/mL.

Cp.	% Reduction of Bacteriophage Phi X174 Virus	% Reduction of Adenovirus Type 7	Cp.	% Reduction of Bacteriophage Phi X174 Virus	% Reduction of Adenovirus Type 7
**1**	70 ± 2	33.3 ± 2	**9**	66.7 ± 3	10 ± 4
**2**	50 ± 2	20 ± 3	**10**	40 ± 3	16.7 ± 2
**3**	50 ± 1	0	**11**	56.7 ± 4	10 ± 4
**4**	10 ± 1	20 ± 3	**12**	20 ± 1	0
**5**	10 ± 4	0%	**13**	30 ± 1	0
**6**	30 ± 2	50 ± 2	**14**	20 ± 4	10 ± 1
**7**	0	0	**15**	20 ± 3	0
**8**	30 ± 3	40 ± 3	**16**	20 ± 4	50 ± 4

**Table 3:** The CC_50_ of the tested compounds against adenovirus type 7 on Hep-2 cell line (µg/mL) using MTT assay.

Comp.	CC_50_	Comp.	CC_50_
**1**	142 ± 1.2	**9**	198 ± 2.5
**2**	123 ± 2.7	**10**	140 ± 3.1
**3**	203 ± 1.6	**11**	193 ± 2.4
**4**	160 ± 1.8	**12**	68 ± 2.3
**5**	118 ± 1.6	**13**	94 ± 3.4
**6**	133 ± 1.1	**14**	174 ± 2.2
**7**	148 ± 2.9	**15**	106 ± 3.3
**8**	108 ± 1.9	**16**	144 ± 3.2

**Table 4:** Molecular docking interactions between selected compounds and the target (the bacteriophage PhiX174 (PDB ID: 2BPA)).

Cp.	Ligand	Receptor	Interaction	Distance Å	E (kcal/mol)	Scoring (kcal/mol)
**1**	S 27	CG PRO294 (F)	H-acceptor	4.05	−0.3	−5.38013
	S 27	N ASP357 (F)	H-acceptor	4.47	−0.9	
	6-ring	CA ASN168 (F)	pi-H	4.03	−0.6	
	6-ring	NE1 TRP30 (J)	pi-H	4.15	−0.3	
**9**	N 30	N ASP357 (F)	H-acceptor	3.17	−3.5	−7.2709
	6-ring	CD1 LEU 166 (F)	pi-H	4.56	−0.4	
	6-ring	CB LYS167 (F)	pi-H	4.64	−1	
	6-ring	CA ARG28 (J)	pi-H	4.6	−0.4	
	6-ring	NE1 TRP30 (J)	pi-H	3.75	−0.3	
**11**	C 4	O LYS167 (F)	H-donor	3.44	−0.3	−6.6476
	N 25	NZ LYS23 (J)	H-acceptor	3.65	−0.8	
	6-ring	CA ARG28 (J)	pi-H	4.67	−0.5	
	6-ring	NE1 TRP30 (J)	pi-H	3.92	−0.4	
	6-ring	NE1 TRP30 (J)	pi-H	3.83	−0.5	

## Data Availability

Not applicable.

## References

[1] Abuelizz H.A., Al-Salahi R. (2023). Significant Pharmacological Activities of Benzoquinazolines Scaffold. Pharmacol. Rep..

[2] Al-Salahi R., Anouar E.H., Marzouk M., Abuelizz H.A. (2019). Anti-HAV Evaluation and Molecular Docking of Newly Synthesized 3-Benzyl(Phenethyl)Benzo[g]Quinazolines. Bioorg. Med. Chem. Lett..

[3] Abuelizz H.A., Marzouk M., Bakheit A.H., Al-Salahi R. (2020). Investigation of Some Benzoquinazoline and Quinazoline Derivatives as Novel Inhibitors of HCV-NS3/4A Protease: Biological, Molecular Docking and QSAR Studies. RSC Adv..

[4] Al-Salahi R., Abuelizz H.A., Ghabbour H.A., El-Dib R., Marzouk M. (2016). Molecular Docking Study and Antiviral Evaluation of 2-Thioxo-Benzo[g]Quinazolin-4(3H)-One Derivatives. Chem. Cent. J..

[5] Maddry J.A., Chen X., Jonsson C.B., Ananthan S., Hobrath J., Smee D.F., Noah J.W., Noah D., Xu X., Jia F. (2011). Discovery of Novel Benzoquinazolinones and Thiazoloimidazoles, Inhibitors of Influenza H5N1 and H1N1 Viruses, from a Cell-Based High-Throughput Screen. J. Biomol. Screen..

[6] Wright B.W., Logel D.Y., Mirzai M., Pascovici D., Molloy M.P., Jaschke P.R. (2021). Proteomic and Transcriptomic Analysis of Microviridae ΦX174 Infection Reveals Broad Upregulation of Host Escherichia Coli Membrane Damage and Heat Shock Responses. mSystems.

[7] Michel A., Clermont O., Denamur E., Tenaillon O. (2010). Bacteriophage PhiX174′s Ecological Niche and the Flexibility of Its Escherichia Coli Lipopolysaccharide Receptor. Appl. Environ. Microbiol..

[8] Hafenstein S., Fane B.A. (2002). ΦX174 Genome-Capsid Interactions Influence the Biophysical Properties of the Virion: Evidence for a Scaffolding-like Function for the Genome during the Final Stages of Morphogenesis. J. Virol..

[9] Bernal R.A., Hafenstein S., Esmeralda R., Fane B.A., Rossmann M.G. (2004). The ΦX174 Protein J Mediates DNA Packaging and Viral Attachment to Host Cells. J. Mol. Biol..

[10] Sun L., Young L.N., Zhang X., Boudko S.P., Fokine A., Zbornik E., Roznowski A.P., Molineux I.J., Rossmann M.G., Fane B.A. (2014). Icosahedral Bacteriophage ΦX174 Forms a Tail for DNA Transport during Infection. Nature.

[11] Jaschke P.R., Dotson G.A., Hung K.S., Liu D., Endy D. (2019). Definitive Demonstration by Synthesis of Genome Annotation Completeness. Proc. Natl. Acad. Sci. USA.

[12] Bosch A. (1998). Human Enteric Viruses in the Water Environment: A Minireview. Int. Microbiol..

[13] Contreras-Coll N., Lucena F., Mooijman K., Havelaar A., Pierzo V., Boque M., Gawler A., Höller C., Lambiri M., Mirolo G. (2002). Occurrence and Levels of Indicator Bacteriophages in Bathing Waters throughout Europe. Water Res..

[14] Valentine C.R., Delongchamp R.R., Pearce M.G., Rainey H.F., Dobrovolsky V.N., Malling H.V., Heflich R.H. (2010). In Vivo Mutation Analysis Using the ΦX174 Transgenic Mouse and Comparisons with Other Transgenes and Endogenous Genes. Mutat. Res. Rev. Mutat. Res..

[15] Shkoporov A.N., Hill C. (2019). Bacteriophages of the Human Gut: The “Known Unknown” of the Microbiome. Cell Host Microbe.

[16] Hamdy N.A., El-Senousy W.M. (2013). Synthesis and Antiviral Evalution of Some Novel Pyrazoles and Pyrazolo [3, 4-d] Pyridazines Bearing 5, 6, 7, 8-Tetrahydronaphthalene. Acta Pol. Pharm..

[17] Qiu F., Shen X., Zhao M., Zhao L., Duan S., Chen C., Qi J.-J., Li G., Wang L., Feng Z. (2018). A Triplex Quantitative Real-Time PCR Assay for Differential Detection of Human Adenovirus Serotypes 2, 3 and 7. Virol. J..

[18] Carballal G., Videla C., Misirlian A., Requeijo P.V., Aguilar M.d.C. (2002). Adenovirus Type 7 Associated with Severe and Fatal Acute Lower Respiratory Infections in Argentine Children. BMC Pediatr..

[19] Al-Salahi R.A., Al-Omar M.A., Alswaidan I., Marzouk M., El-Senousy W.M., Amr A.E.-G.E. (2015). Antiviral Activities of Some Synthesized Methylsulfanyltriazoloquinazoline Derivatives. Res. Chem. Intermed..

[20] Abuelizz H.A., Marzouk M., Bakheit A.H., Awad H.M., Soltan M.M., Naglah A.M., Al-Salahi R. (2020). Antiproliferative and Antiangiogenic Properties of New VEGFR-2-Targeting 2-Thioxobenzo [g] Quinazoline Derivatives (In Vitro). Molecules.

[21] Al-Salahi R., Moustapha M.E., Abuelizz H.A., Alharthi A.I., Alburikan K.A., Ibrahim I.T., Marzouk M., Motaleb M.A. (2018). Radioiodination and Biodistribution of Newly Synthesized 3-Benzyl-2-([3-Methoxybenzyl] Thio) Benzo [g] Quinazolin-4-(3H)-One in Tumor Bearing Mice. Saudi Pharm. J..

[22] Al-Salahi R., Taie H.A.A., Bakheit A.H., Marzouk M., Almehizia A.A., Herqash R., Abuelizz H.A. (2019). Antioxidant Activities and Molecular Docking of 2-Thioxobenzo[g]Quinazoline Derivatives. Pharmacol. Rep..

[23] Al-Salahi R., Ahmad R., Anouar E., Iwana Nor Azman N.I., Marzouk M., Abuelizz H.A. (2018). 3-Benzyl(Phenethyl)-2-Thioxobenzo[g]Quinazolines as a New Class of Potent α-Glucosidase Inhibitors: Synthesis and Molecular Docking Study. Future Med. Chem..

[24] Abuelizz H.A., Marzouk M., Bakhiet A., Abdel-Aziz M.M., Ezzeldin E., Rashid H., Al-Salahi R. (2021). In silico study and biological screening of benzoquinazolines as potential antimicrobial agents against methicillin-resistant Staphylococcus aureus, carbapenem-resistant Klebsiella pneumoniae, and fluconazole-resistant Candida albicans. Microb. Pathog..

[25] Al-Salahi R., Abuelizz H.A., Wadi M., El Dib R.A., Alotaibi M.A., Marzouk M. (2016). Antimicrobial Activity of Synthesized 2-Methylthiobenzo[g][1, 2, 4]Triazolo [1, 5-a] Quinazoline Derivatives. Med. Chem..

[26] Al-Salahi R., El Dib R.A., Marzouk M. (2015). Synthesis and in Vitro Cytotoxicity Evaluation of New 2-Thioxo-Benzo[g]Quinazolin-4(3h)-One Derivatives. Heterocycles.

[27] Al-Salahi R., Abuelizz H.A., El Dib R., Marzouk M., Alshammari M.B. (2017). Antimicrobial Activity of New 2-Thioxo-Benzo[g]Quinazolin-4(3H)-One Derivatives. Med. Chem..

[28] Simões C.M.O., Amoros M., Girre L. (1999). Mechanism of Antiviral Activity of Triterpenoid Saponins. Phytother. Res. Int. J. Devoted Pharmacol. Toxicol. Eval. Nat. Prod. Deriv..

[29] Walum E., Stenberg K., Jenssen D. (1990). Understanding Cell Toxicology.

[30] Schmidtke M., Knorre C., Blei L., Stelzner A., Birch-Hirschfeld E. (1998). Penetration and Antiviral Activity of Coxsackievirus B3 (CVB3)-Specific Phosphorothioate Oligodeoxynucleotides (PS-ODN). Nucleosides Nucleotides.

[31] Mosmann T. (1983). Rapid colorimetric assay for cellular growth and survival: Application to proliferation and cytotoxicity assays. J. Immunol. Methods.

[32] El-Baz F.K., El-Senousy W.M., El-Sayed A.B., Kamel M.M. (2013). In vitro antiviral and antimicrobial activities of algal extract of *Spirulina platensis*. J. Appl. Pharm. Sci..

